# RETRACTED: Dopelt et al. (2025). What Drives the Non-Medical Use of Stimulants Among College Students? The Role of Self-Efficacy and Attitudes: A Cross-Sectional Study of Israeli Undergraduates. *European Journal of Investigation in Health, Psychology and Education*, *15*(7), 141

**DOI:** 10.3390/ejihpe16070107

**Published:** 2026-07-22

**Authors:** Keren Dopelt, Shiran Bord, Nourit Houminer-Klepar

**Affiliations:** 1 Department of Public Health, Ashkelon Academic College, Ashkelon 78211, Israel; nhouminer@gmail.com; 2 Department of Health Systems Management, The Max Stern Yezreel Valley College, Emek Yezreel 19306, Israel; shiranb@yvc.ac.il

The journal retracts the article titled “What Drives the Non-Medical Use of Stimulants Among College Students? The Role of Self-Efficacy and Attitudes: A Cross-Sectional Study of Israeli Undergraduates” (Dopelt et al., 2025), cited above.

Following publication, concerns were raised with the Editorial Office regarding the accuracy of certain cited sources in this article (Dopelt et al., 2025).

In accordance with standard journal procedures, the Editorial Office and Editorial Board conducted an investigation that confirmed the presence of a number of citations that either do not appear to exist or lack appropriate relevance to the context of the research. As a result, the Editorial Board has lost confidence in the reliability of the findings and decided to retract this publication (Dopelt et al., 2025), as per MDPI’s retraction policy (https://www.mdpi.com/ethics#_bookmark30, accessed on 11 March 2026).

This retraction was approved by the Editor-in-Chief of the *European Journal of Investigation in Health, Psychology and Education*.

The authors have been informed of this retraction and have indicated their agreement with this decision.

## References

[B1-ejihpe-16-00107] Dopelt K., Bord S., Houminer-Klepar N. (2025). RETRACTED: What drives the non-medical use of stimulants among college students? The role of self-efficacy and attitudes: A cross-sectional study of Israeli undergraduates. European Journal of Investigation in Health, Psychology and Education.

